# Prevalence of symptoms of burnout syndrome in primary health care professionals

**DOI:** 10.47626/1679-4435-2023-813

**Published:** 2023-04-18

**Authors:** Maria Luiza Fucuta-de-Moraes, Jéssica Cristina Ruths

**Affiliations:** 1 Medicina, Universidade Federal do Paraná, Toledo, PR, Brazil

**Keywords:** professional burnout, psychological burnout, primary health care, esgotamento profissional, esgotamento psicológico, atenção primária à, saúde

## Abstract

**Introduction:**

Burnout syndrome results from a response to chronic work stress and is responsible for causing symptoms related to three dimensions: emotional exhaustion, reflecting work overload; depersonalization, characterized by professional detachment and cynicism; and reduced professional accomplishment, related to low productivity at work. Burnout is often associated with jobs that require professionals to have direct contact with users, such as health professionals. Primary Health Care is the assistance level that greatest contact with the community and requires teamwork, thus exposing workers to potential psychosocial stressors.

**Objectives:**

To identify the prevalence of symptoms of burnout syndrome among Primary Health Care professionals in Toledo, state of Paraná, Brazil.

**Methods:**

This was a descriptive, quantitative, cross-sectional study. A sociodemographic questionnaire and the Maslach Burnout Inventory, Human Services Survey, were used to assess the outcomes.

**Results:**

The prevalence of high risk for the development of burnout syndrome was 10.6%, and, when dimensions were evaluated separately, it was found that 29.8, 52.1 and 22.3% of participants showed a high level of symptoms in the dimensions emotional exhaustion, reduced professional accomplishment, and depersonalization, respectively. Previous use of psychiatric medication due to another condition had a significant correlation with high risk for burnout.

**Conclusions:**

The results of this research corroborated other similar studies, contributing to knowledge on the subject in a region of the state of Paraná where there was still no research on the syndrome.

## Abstract (pt)

**Introdução:**

A síndrome de *burnout* é decorrente de uma resposta ao estresse laboral crônico, responsável por gerar sintomas contidos em três dimensões: exaustão emocional, refletindo a sobrecarga do trabalho; despersonalização, caracterizada pelo distanciamento profissional e cinismo; e baixa realização profissional, relacionada à baixa produtividade do trabalhador. Frequentemente, o *burnout* está associado a cargos que exigem do profissional contato direto com os usuários, como os profissionais de saúde. A Atenção Primária em Saúde é o nível assistencial de maior contato com a comunidade e requer trabalho em equipe, expondo o trabalhador a potenciais estressores psicossociais.

**Objetivos:**

Este estudo objetivou identificar a prevalência dos sintomas da síndrome de *burnout* nos profissionais de saúde da Atenção Primária em Saúde do município de Toledo, estado do Paraná.

**Métodos:**

Tratou-se de um estudo descritivo de delineamento transversal, com análise quantitativa. Um questionário sociodemográfico e o Maslach Burnout Inventory, versão Human Services Survey, foram usados para avaliar o desfecho.

**Resultados:**

A prevalência de alto risco para o desenvolvimento da síndrome de *burnout* foi de 10,6%, e as dimensões, quando avaliadas separadamente, demonstraram que 29,8, 52,1 e 22,3% dos participantes obtiveram alto nível de sintomas nas dimensões exaustão emocional, baixa realização profissional e despersonalização, respectivamente. O uso prévio de medicação psiquiátrica por outra afecção teve correlação significativa com o alto risco da síndrome.

**Conclusões:**

Os resultados desta pesquisa corroboraram outros estudos semelhantes, contribuindo para o conhecimento sobre o assunto em uma região do Paraná onde ainda não havia pesquisas sobre a síndrome.

## INTRODUCTION

Balance between humans and nature depends on their synchrony with the environment. An instability in this relationship may lead to dysfunctions in individual’s physical, mental, and social processes. In the context of burnout syndrome, work is the environmental factor that causes this situation when working conditions do not suit worker’s skills and needs.^1^

Burnout syndrome, also known as burnout or professional burnout, is defined as a response resulting from chronic worker’s exposure to labor stressors, so that the adaptive mechanisms developed to cope with this scenario are no longer sufficient. The syndrome includes three characteristic dimensions: emotional exhaustion, depersonalization, and reduced professional accomplishment or professional inefficiency.^1-3^

Emotional exhaustion is the key dimension of the syndrome, being thus essential but not sufficient to characterize it. This symptom consists of a response to job overload, in which workers lack energy to cope with everyday work problems. It is the most reported and observed dimension in studies, and needs to be inserted into the occupational context to be part of the phenomenon called burnout syndrome.^1-3^

Depersonalization emerges as a self-protection mechanism when individuals perceive the need to reduce their activities in order to care for their mental health. However, when this ideation goes beyond reasonable, professionals fail to provide a humanized service, making little effort during work activities and time dedicated to them, which generates cynical individuals who are cognitively and emotionally disconnected from their job.^1-3^

The last dimension, characterized by reduced professional accomplishment and professional inefficacy, is considered by some as the result or combination of the other two dimensions. It refers to incompetence and lack of work productivity, which results in a feeling of guilt for having chosen the wrong career, which lead workers to have a negative self-image.^1-3^

Maslach et al.^2^ assume that individual and especially external factors are related to phenomenon. Thus, workload, employee recognition, availability of resources, respect for workers’ autonomy, and institution’s general organization are some factors that influence the way individuals deal with their job demands and may thus be closely related to burnout syndrome.^2^

Therefore, it is observed that patients with this syndrome are usually workers experiencing a derangement in some of these factors. They are often professionals who need to have direct and constant contact with other people, being sometimes exposed to intense pressure and stress, such as professionals in the areas of health care, education, and safety.^2,3^

Furthermore, studies point out that, among health professionals, those working in Primary Health Care (PHC) services have the highest prevalence of burnout syndrome.^4-6^ This situation occurs because their work involves direct and continuous contact with patients, which means they often need to deal with complex demands, in order to provide comprehensive health care. This generates a continuous exposure to psychosocial stressors.^7^

Furthermore, PHC provision depends on the availability of financial resources and infrastructure in the workplace for the provision of an efficient service to the community, which may lead to frustration and dissatisfaction among health professionals when there is scarcity and unsanitary conditions, because these professionals aim to provide a comprehensive service to the community.^7^

There is a need to study professional burnout, since it is associated with reduced productivity and increased levels of absenteeism, job turnover, and occupational accidents, possibly resulting in financial losses to the institution and to workers themselves.^7^ Data provided by investigations may be used to evaluate factors associated with the development of burnout syndrome and what of these factors can be prevented or not.

Therefore, the present study aimed to analyze the prevalence of symptoms of burnout syndrome among PHC professionals in the municipality of Toledo, state of Paraná (PR), Brazil. We hope the results of this research contribute with relevant data for managers and workers, in order to help in the development of health prevention public policies.

## METHODS

This was a quantitative, descriptive, cross-sectional study seeking to characterize the occurrence of symptoms of burnout syndrome among PHC professionals in Toledo, PR, Brazil, and correlate these symptoms with the sociodemographic profile of the study population.

The study was conducted in 11 Basic Health Units (BHUs) located in Toledo, PR, Brazil, and two instruments were used for data collection. The first one was a sociodemographic questionnaire including the variables sex, age, occupation, weekly working hours, number of employment relationships, monthly income, and use of psychiatric medication. The second one was the Maslach Burnout Inventory (MBI), version Human Services Survey (HSS) specific for health professionals, through which symptoms of emotional exhaustion, depersonalization, and reduced professional accomplishment were assessed.

The MBI-HSS was designed by Christina Maslach & Susan Jackson,^8^ translated and validated to Portuguese by Lautert.^1^ It consists of 22 items, of which nine were related to emotional exhaustion; five, to depersonalization; and eight, to reduced professional accomplishment. Each item refers to a statement that reflects the respondent’s feelings regarding their occupation. Answers were based on a 5-point Likert scale, in which 1 means “never;” 2, “a few times per year;” 3, “a few times per month;” 4, “a few times per week;” and 5, “everyday,” in order to assess the frequency with which respondents identified with the feeling expressed in each statement.^8-10^

Considering the high prevalence of burnout syndrome among PHC professionals who have direct contact with patients, this study included physicians, nurses, nursing technicians, and nursing assistants who had been working at BHUs in Toledo, PR, Brazil, for more than 6 months, who had weekly working hours of at least 20 hours, and who completed at least 75% of the MBI-HSS. Workers who had been absent from work due to health reasons or intentional work leave for the entire data collection period were excluded from the sample, as well as those who refused to participate in the research.

The prevalence of symptoms of burnout syndrome was assessed using the scores obtained in each MBI dimension, classified according to the cutoff scores shown in Table 1. A high level in any dimension indicated the presence of symptoms for this variable, whereas medium or low levels were associated with few or no symptoms, respectively.^11^

**Table 1:** Scoring system to assess the level of symptoms of burnout syndrome^11^

Dimensions	Scoring system		
	High level	Medium level	Low level
Emotional exhaustion	≥ 27	17-26	≤ 15
Depersonalization	≥ 13	7-12	≤ 6
Reduced professional accomplishment	≤ 30	31-38	≥ 39

Workers were considered to have a high levels of symptoms in the dimensions emotional exhaustion and depersonalization when their scores were high. In reduced professional accomplishment, lower scores indicated a high level of symptoms, since they show that participant’s score was low for questions addressing professional accomplishment. Similarly, lower scores for emotional exhaustion and depersonalization and higher scores for reduced professional accomplishment indicated low level of symptoms.^11^

An analysis of questionnaires enabled to identify participants’ scores for the three dimensions, which were jointly categorized into situations of high, moderate, or low risk for the development of burnout syndrome. High levels of symptoms in the three dimensions indicated high risk for burnout syndrome, i.e., high levels of emotional exhaustion, high levels of depersonalization, and reduced professional accomplishment. The presence of high levels of symptoms in two dimensions was defined as moderate risk for burnout, and the presence of high levels of symptoms in only one dimension, or no high levels of symptoms in any dimension was considered as low risk for burnout syndrome.^11^

The association between sociodemographic variables and burnout syndrome was tested using the Spearman’s rho correlation test for the variables working hours, age, number of employment relationships, and monthly income, and using the chi-square test of association for the variable use of psychiatric medication. Calculations were made using the SPSS, software, version 25.

The present study complied with the ethical principles of guidelines and standards regulating research involving human beings set for in Resolution 466/12 of the Brazilian National Health Council.^12^ Furthermore, the study was approved by Research Ethics Committee of Universidade Federal do Paraná (UFPR), under process number 3683180/2019. There is no conflict of interest related to this study.

## RESULTS

Overall, 109 workers participated in this study, of which 15 were excluded from the sample due to incorrect or incomplete completion of the instrument; thus, 94 correctly completed questionnaires (86.23%) remained in the study.

Most participants were female (76.14%) and were younger than 40 years of age (71.00%), and more than a half was aged from 30 to 39 years (59%). The predominant professional categories were nursing technicians (42.05%) and nurses (31.82%). The most prevalent working hours was from 40 to 60 hours per week (62.07%), since 80.23% of participants had only one employment contract in PHC. Almost half of the workers (44.58%) had a monthly income lower than three minimum wages. Moreover 17.05% answered affirmatively to the question on use of psychiatric medication.

The study found suggestive high risk for burnout syndrome in 10.6% (Table 2) of participants, of which six belonged to the nursing staff, two to the medical staff, and one to the oral health staff. Most participants (71.30%) were classified as having low risk for the development of burnout syndrome.

**Table 2:** Prevalence of symptoms of burnout syndrome according to risk classification

Classification of risk for burnout syndrome	n	%
High	10	10.6
Moderate	17	18.1
Low	67	71.3

When dimensions were assessed separately, it was found that 29.8% of participants had a high level of symptoms of emotional exhaustion; 52.1%, of reduced professional accomplishment; and 22.3%, of depersonalization. However, there was a predominance of medium level of symptoms for the three variables (Table 3).

**Table 3:** Prevalence of high, medium, and low level of symptoms of burnout syndrome for the dimensions emotional exhaustion, reduced professional accomplishment, and depersonalization

Classification	Emotional exhaustion		Reduced professional accomplishment		Depersonalization	
	n = 94	%	n = 94	%	n = 94	%
High	28	29.8	49	52.1	21	22.3
Medium	51	54.3	39	41.5	50	53.2
Low	15	16.0	6	6.4	23	24.5

When data regarding gender were separately related to dimensions, it was observed that both men and women showed a similar prevalence of high levels of emotional exhaustion and reduced professional accomplishment, 28.3 and 28.5%, respectively. Conversely, men had a higher prevalence for depersonalization (38.09%), more than twice as that found among women (17.90%).

Although seven participants out of the ten classified as at high risk for burnout syndrome were younger than 40 years, the prevalence for high levels of symptoms in each separate dimension was similar among age groups. The most relevant difference was found in reduced professional accomplishment, a variable in which most (50.84%) of younger participants was classified as having a high level of symptoms, whereas most (58.30%) of the older individuals had a mean level of symptoms.

In terms of professional category, the prevalence of high risk for burnout syndrome was 11.7% among physicians and 9.23% among the nursing staff. One member of the oral health staff had a high risk for burnout, out of the six participants belonging to this staff. When each dimension was compared separately, professional categories exhibited similar values, and it was worth highlighting that more than 90% of physicians and nursing professionals had medium to high levels of symptoms for reduced professional accomplishment.

There was no significant difference between the groups with regard to working hours and number of employment relationships. It was observed that only three professionals worked more than 60 hours per week, and all of them were classified as having low risk for burnout syndrome. Furthermore, those who worked from 40 to 60 hours per week showed a slightly higher prevalence of emotional exhaustion (31.40%) compared to those who worked less than 40 hours (27.50%).

The test for association between sociodemographic variables and MBI scores revealed that only the variable use of psychiatric medication was significantly associated (p = 0.001). Half of participants at high risk for burnout syndrome were using psychiatric medication.

## DISCUSSION

Despite the scarcity of studies on burnout syndrome, there has been an increasing scientific interest in the subject, since this syndrome is associated with negative consequences not only to worker’s physical and mental health but also to institution’s socioeconomic scenario. In this context, in order to establish trends and statistics about burnout syndrome, it was added to International Classification of Diseases, 11th revision (ICD-11) of the World Health Organization (WHO), as QD85.^13,14^

The instrument used in our research was MBI-HSS, the most renowned and used questionnaire for studies about this syndrome. The MBI provides separate scores for each dimension, based on the frequency with which the respondent experiences the described symptoms.^8^ The present research adopted the definition of Maslach^2^ and of Ramirez et al.,^15^ which states burnout syndrome is present when the three dimensions show high levels of symptoms, so that high scores for emotional exhaustion and depersonalization associated with low scores for professional accomplishment suggest the presence of the phenomenon.^5,16^

The predominant profile of study participants consisted of female health professionals aged from 30 to 39 years who worked in the nursing staff of PHC services in Toledo, PR, Brazil, and who had the BHU as their only employment contract. The higher proportion of females, also reported in other studies,^16,17^ may be explained by the fact that the nursing staff was historically composed of women, socially conditioned to the role of caregivers.^18^

The prevalence of burnout syndrome found in this research was 10.6%, similar to that observed in other studies conducted in the Brazilian PHC network: 11.2% in Rio de Janeiro, according to Porciuncula et al.;^6^ 7% in Sergipe, according to Silva et al.;^19^ 6.9% in Rio Grande do Sul, according to Trindade et al.;^20^ and 9.5% in São Paulo, according to Poletto et al.^21^

Most professionals (71.30%) had low risk for the development of burnout syndrome. However, there was a predominance of medium level of symptoms for emotional exhaustion and depersonalization and of high level symptoms for reduced professional accomplishment. Noteworthy, only 6.4% of participants expressed professional accomplishment, according to MBI scores. Furthermore, there were remarkably high levels of symptoms of emotional exhaustion and depersonalization, 29.8 and 22.3%, respectively.

Professional accomplishment involves worker’s satisfaction, especially with organizational factors. In other words, it is related to autonomy and organization policies, hierarchy of positions, provision of resources and benefits to employees, among other factors.^22^ Several studies indicate that poor infrastructure, teamwork difficulties, shortage of resources, and lack of recognition are common in the PHC setting; hence, all these organizational characteristics that may be related to the reduced professional accomplishment found in this context.^19,23,24^

Similarly, direct exposure to the reality of the community where professionals work, conflicts in interpersonal relationships with health professionals and patients, exposure to physical and verbal violence, and high work demands are aspect that justify increased emotional exhaustion and depersonalization in the PHC scenario, which may have contributed to the high levels of symptoms for these dimensions.^23^

As well as in the studies by Trindade et al.^20^ and Porciuncula et al.,^6^ occupation was not directly correlated with risk for the development of the syndrome. Physicians were the professional category that had the highest percentage of high risk (11.70%), followed by nurses, with a 9.23% prevalence. Physicians were found to have a high prevalence of burnout in many study, which may explained by the growing pressure on this professional category for productivity, increased number of consultations, and curative measures, in addition to the feeling of frustration and impotence when facing more complex clinical situations.^4,5,19^

However, previous use of psychiatric medication due to another condition had a significant correlation with the development of symptoms of burnout syndrome. This was consistent with the study by Martins et al.,^7^ which found that use of hypnotics, anxiolytics, and antidepressants was associated with greater risk for burnout syndrome.

Therefore, it is possible to question whether the professionals taking these drugs were treating manifestations of burnout syndrome as another disease, or another disease could be causing symptoms similar to those of burnout.

Depression is known to be one of the differential diagnoses of professional burnout, since it presents similar symptoms, such as emotional exhaustion, although its etiology not necessarily occupational. Burnout syndrome results from organizational and interpersonal relationships, whereas depression is an array of thoughts and emotions that exactly affect these interpersonal relationships, but is not necessarily caused by individual’s occupation.^25^

According to the literature, among individual factors, age is the most related to burnout syndrome, with workers younger than 40 years being the most prone to develop it.^2^ This may be explained by the fact that younger individuals have more expectations regarding their careers and thus become more disappointed when the job does not ensure the fulfillment of their desires and expectations.^20^

In line with studies by Trindade et al.^20^ and Silva et al.,^19^ our research showed that most individuals at high risk for burnout syndrome were young, but there was significant correlation. The dimension with the greatest discrepancy between the two age groups was reduced professional accomplishment, with half of those younger than 40 years had high levels of symptoms, compared to 37.5% of older participants. This may result from unreached expectations, generating a situation of reduced professional accomplishment.

Gil-Monte^18^ states that there are differences between genders regarding the variable depersonalization, so that men tend to obtain higher scores than women. In the present research, there was no significant association between gender and prevalence of high risk for burnout syndrome; however, the fact that most of the study population comprised women may have influenced this analysis.

Consistent with the above author, both genders obtained similar scores for emotional exhaustion and reduced professional achievement in our study; nevertheless, the prevalence of high level of depersonalization symptoms was more than twice higher among men (38.09%) compared to women (17.90%). Gil-Monte^18^ relates this situation with hardness and emotional indifference in interpersonal relationships that characterize the male sex.

## CONCLUSIONS

We consider that the present study has internal validity, since it analyzed a representative sample in the assessed context and used a validated instrument. Therefore its results may reveal the situation of PHC professionals from Toledo, PR, Brazil. Knowing that the PHC services adopt similar health policies in other municipalities and face similar problems, our results are supposed to be used to support public health policies beyond the local level.
